# Analysis of causes for poor persistence of CAR-T cell therapy *in vivo*

**DOI:** 10.3389/fimmu.2023.1063454

**Published:** 2023-01-25

**Authors:** Yingjie Kong, Ling Tang, Yong You, Qing Li, Xiaojian Zhu

**Affiliations:** ^1^ Institute of Hematology, Union Hospital, Tongji Medical College, Huazhong University of Science and Technology, Wuhan, China; ^2^ Department of Hematology, Wuhan No.1 Hospital, Wuhan, China; ^3^ Department of Hematology, Tongji Hospital, Tongji Medical College, Huazhong University of Science and Technology, Wuhan, China

**Keywords:** CAR-T cells, relapsed/refractory, the structure of CARs, the proportion of memory CAR-T cells, tumor microenvironment

## Abstract

Chimeric antigen receptor T-cell (CAR-T-cell) therapy has been well researched to date because of its ability to target malignant tumor cells. The most common CAR-T cells are CD19 CAR-T cells, which play a large role in B-cell leukemia treatment. However, most CAR-T cells are associated with relapse after clinical treatment, so the quality and persistence of CAR-T cells need to be improved. With continuous optimization, there have been four generations of CARs and each generation of CARs has better quality and durability than the previous generation. In addition, it is important to increase the proportion of memory cells in CAR-T cells. Studies have shown that an immunosuppressive tumor microenvironment (TME) can lead to dysfunction of CAR-T cells, resulting in decreased cell proliferation and poor persistence. Thus, overcoming the challenges of immunosuppressive molecules and targeting cytokines in the TME can also improve CAR-T cell persistence. In this paper, we explored how to improve the durability of CAR-T cell therapy by improving the structure of CARs, increasing the proportion of memory CAR-T cells and improving the TME.

## 1 Introduction

In recent years, great progress has been made in chimeric antigen receptor T cell (CAR-T cell) therapy for hematological and solid tumors, but patients still experience recurrence after treatment. Poor persistence of CAR-T cells *in vivo* is an important cause of relapse (1). This review summarizes three factors limiting why CAR-T cells persistence *in vivo*: the structure of CARs, the proportion of memory CAR-T cells and the TME. The CARs structure of CAR-T cells has undergone four generations of evolution. Second-generation CARs include additional costimulatory domains that are lacking in first-generation CARs, third-generation CARs have two costimulatory domains, and fourth-generation CARs have both costimulatory domains and other domains that can regulate cytokines or other molecules. With the structural additions of each generation of CARs, the survival and persistence of CAR-T cells *in vivo* has improved (2–11). Studies have shown that the higher the percentage of memory T cells, the more durable the T cells are in the vivo (12). Therefore, it is essential to improve the proportion of memory CAR-T cells, which can be increased in four ways: preventing T cell differentiation (13–17); reprogramming terminally differentiated T cells (18, 19); shortening the culture time of CAR-T cells (20) and delaying the senescence of CAR-T cells (21). In addition, immune checkpoint molecules and certain cytokines in the TME can also shorten the lifespan and reduce the function of T cells (22–24).

## 2 The structure of CARs can influence CAR-T cell proliferation and persistence

**Figure 1:**
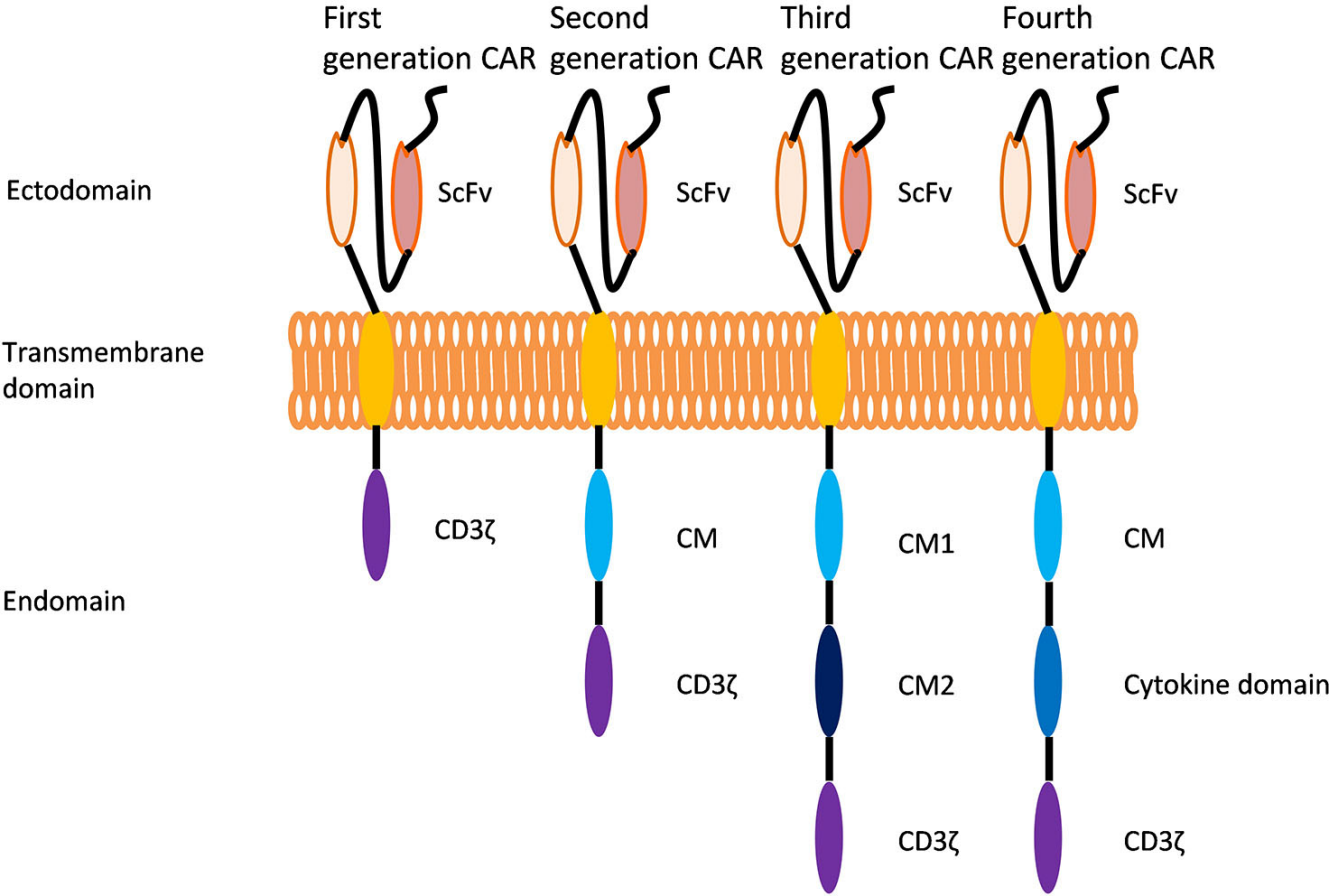
Evolution of chimeric antigen receptors (CARs). Each CAR generation contains an extracellular single-stranded variable region (scFv), an intracellular CD3 domain (CD3ζ) and a T-cell receptor transmembrane domain. The first generation of CARs has only the CD3 domain in the cell. The second generation of CARs includes the CD3 domain and the costimulatory domain (CM). Developed on the basis of second-generation CARs, the third generation of CARs has two different costimulatory domains in the cell. Developed based on the structure of second-generation of CARs, fourth-generation of CARs have an additional intracellular domain that regulates the expression of cytokines or other costimulatory molecules.

CAR-T cell therapy consists of editing T cells to express specific CARs and reinjecting the cells expanded *in vitro* into the patient to eradicate tumors (4). A CAR is a receptor consisting of three main portions: an extracellular antigen recognition domain, a transmembrane domain, and an intracellular signaling domain (25) (**Figure 1**).

### 2.1 First-generation CARs of CAR-T cells

First-generation CARs contain a single-chain variable fragment (scFv) that is linked to the intracellular signaling domain of CD3ζ, providing the signal that is necessary to activate T cells; however, these CARs only provide the T cell priming signal and have no costimulatory molecules. The lack of costimulatory molecules results in low activation and antitumor effects of T cells *in vivo* (2, 3)..

### 2.2 Second-generation CARs of CAR-T cells

**Figure 2:**
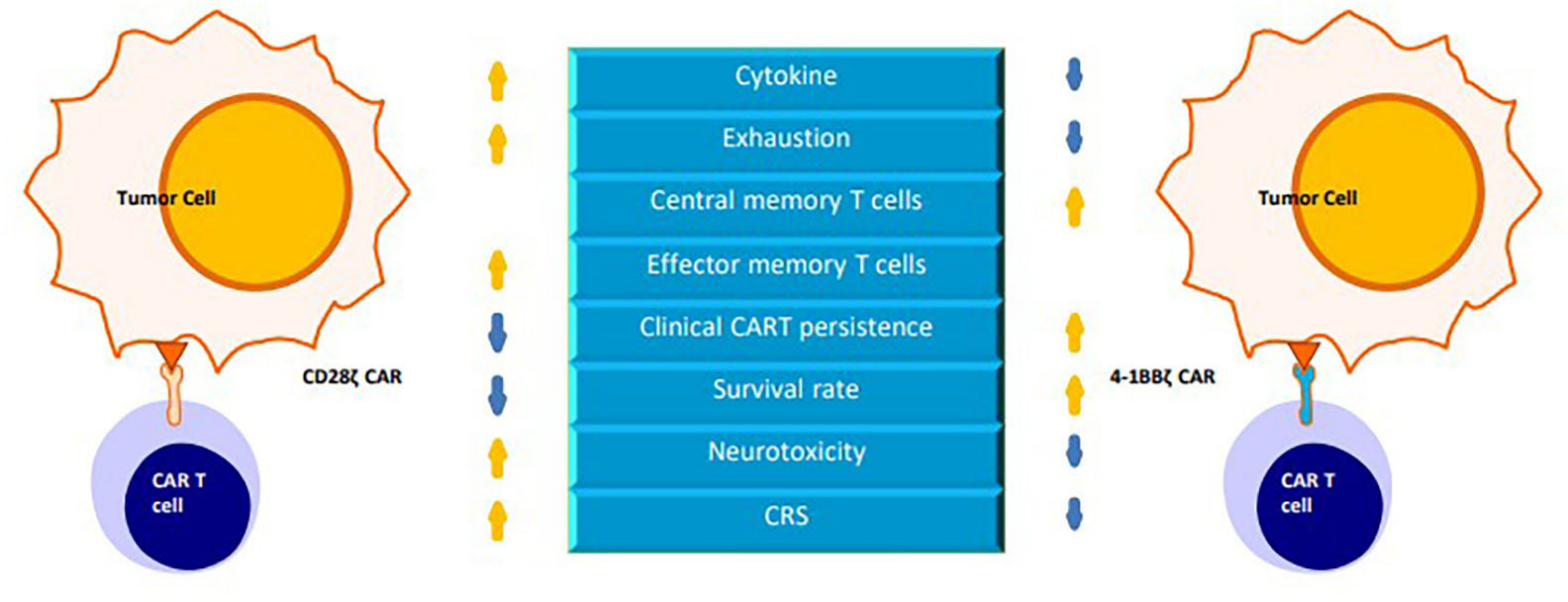
A comparison of chimeric antigen receptors containing CD28 versus 4-1BB costimulatory domains.

Developed based on first-generation CARs, second-generation CARs include a costimulatory domain (CD28, 4-1BB, OX-40, ICOS and CD134) that provides a second signal for T-cell activation (4, 5). The second signal prevents the exhaustion of CAR-T cells and promotes their continued proliferation and cytokine secretion, resulting in increased potency in the killing of target cells *in vivo* (6, 7). This review focuses on the CD28 and 4-1BB costimulatory domains (**Figure 2**).

CD28 is a transmembrane protein and a member of the immunoglobulin superfamily (26). The CD28 costimulatory receptor is expressed on both CD8+ T and CD4+ T cells. The extracellular portion of CD28 binds to the B7 family of ligands [CD80, CD86 (B7-2), and CD275 (B7-H2) (27–29)] to activate intracellular signaling cascades, such as the PI3K, NF-κB,AP-1 and NFAT signaling pathways, which can regulate T-cell proliferation and survival and promote IL-2 production (26, 30, 31).

4-1BB (also known as CD137 and TNFRSF9) is also a transmembrane protein and a member of the tumor necrosis factor receptor superfamily (TNFRSF). 4-1BB is found on antigen-activated CD8+ and CD4+ T cells (32). 4-1BB binds to its sole ligand (4-1BBL) to activate downstream signaling by recruiting TRAF proteins (33).

The CD28 signaling domain represents an “early” costimulatory signal that promotes the proliferation of T cells, accelerates the differentiation of effector memory T (TEM) cells and CD8+ T cells, and leads to increased more IL-2 production. Encoding a fully functional CD28 signaling domain on the CAR polypeptide chain may produce overstimulation, increase the incidence of cytokine release syndrome (CRS), promote T-cell exhaustion, and reduce persistence (33–35)..

The 4-1BB signaling domain is responsible for the “late” costimulatory signal that promotes T cell differentiation into central memory T(TCM) cells, increases the oxidative metabolism and glycolysis of CAR-T cells, enhances T cell persistence and improves the antitumor ability of T cells (34, 36).

Studies showed that the 4-1BB domain in CAR activated ncNF-κB signaling in human T cells, thereby increasing the expansion and the survival of 4-1BBζ CAR-T cells *in vitro* (37).

In preclinical studies of CAR-T cells in pre-B cell acute lymphoblastic leukemia (pre-BALL) mice, compared with CD28 costimulation or CD3ζ signaling alone, 4-1BB costimulation of CAR-T cells improved the survival of tumor-bearing mice (7, 38).

In relapsed or refractory B-cell acute lymphoblastic leukemia (r/r B-ALL), 4-1BB-based CAR-T cells have been shown to show higher antitumor efficacy, longer persistence, and fewer serious adverse events than CD28 CAR-T cells (39).

It has been shown that CAR-T cells can live approximately 30 days in patients who are treated with CD28ζ CAR-T cells, while CAR-T cells can persist for more than 4 years in those who are treated with 4-1BBζ CAR-T cells (40–42).

These results indicate that compared to CD28ζCAR-T cells, 4-1BBζCAR-T cells are more persistent but have weaker killing effects *in vivo*. To improve the shortcomings of both CAR-T cells, studies have been conducted to integrate the two costimulatory molecules into the structure of CARs, **leading to the development of** third-generation CARs of CAR-T cells.

### 2.3 Third-generation CARs of CAR-T cells

To increase T cell survival, cytokine production, and antitumor potential, researchers have modified the second-generation CARs by adding another costimulatory domain to the CARs. These modifications include the combination of CD28 and OX40 costimulatory molecules in CD30 CAR-T cells, CD28 and 4-1BB costimulatory molecules in CD19 CAR-T cells and 4-1BB and DAP10 costimulatory molecules in NKG2D(z) CAR-T cells. All of these factors enhance the expansion and persistence of CAR-T cells *in vivo* (8, 43, 44).

Nevertheless, third-generation CARs have risks of off-target effects and excess cytokine production. In addition, there is a lack of clinical data for third-generation CARs. There was clinical efficacy, but the treatment did not cause serious side effects in patients who were not cured (45–47). Hence the fourth-generation of CARs have been generated.

### 2.4 Fourth-generation CARs of CAR-T cells

Fourth-generation CARs which were developed on the basis of second-generation CARs, include not only costimulatory domains but also domains that regulate the expression of cytokines or other costimulatory molecules.

IL-12 is a proinflammatory cytokine with strong tumor inhibitory activity, that not only stimulates T cells to secrete IFN-γ to enhance the cytotoxicity of CAR-T cells, but also limits the activation of regulatory T (Treg) cells, reshaping the TME in an IFN-γ-dependent manner (48). Yingmei Luo, Giulia Agliardi and Hollie J Pegram et al. found administration of CAR-T cells with autocrine IL-12 in the TME significantly improved the killing effect of CAR-T cells and achieved long-lasting antitumor effects (49–51).

IL-18, a member of the IL-1 family of cytokines, enhances T-cell toxicity and promotes IFN-γ secretion. Markus Chmielewski et al. and Mauro P Avanzi et al. developed CD19 CAR-T cells capable of secreting IL-18 for the treatment of solid and hematologic tumors, respectively. The researchers included an anti-CD19 scFv, a CD28 costimulatory domain and a human IL-18 domain. The new CD19 CAR-T cells promoted T cell proliferation and had more CD8+ T cells and TCM cells. Furthermore, compared with second-generation CAR-T cells, they showed better survival in a mouse model (9, 52).

IL-21 plays an important role in the differentiation of memory T cells, and can promote the expansion of CAR-T cells after antigen stimulation, prevent the terminal differentiation of CAR-T cells, and reduce the apoptosis of CAR-T cells. Markley et al. (10) and Harjeet Singh et al. (53) constructed CAR-T cells that can secrete IL-21. The structure of the CARs consisted of an anti-CD19 scFv, a CD28 costimulatory domain and a human IL-21 domain. The research found that the new CD19 CAR-T cells exhibited greater expansion and greater expression of the cytokine IFN-γ when stimulated by tumor cells than conventional CD19 T cells. The cells promoted the differentiation of T cells into a memory phenotype and r had lower levels of apoptosis. CAR-T cells are retained longer in experimental animals.

IL-23 is a two-subunit cytokine composed of the IL-23αp19 and IL12β p40 subunits, both of which are expressed by activated macrophages and dendritic cells (54). In chronic lymphocytic leukemia, IL-23-activated STAT3 contributes to the enhanced function of CAR-T cells (13). IL-23-producing CAR-T cells (P40-TD CAR-T) have been constructed and show higher antitumor ability, increased granzyme B expression and decreased PD-1 expression (55).

The above studies have added domains that can regulate cytokine expression to the structure of second-generation CARs, and the expression of these cytokines increases the persistence of CAR-T cells *in vivo* by improving the TME or promoting the generation of memory T cells. More different cytokine domains can be added to the structure of CARs in the future to increase the persistence of CAR-T cells *in vivo*.

### 2.5 Others

In addition, targeting transcription factors can reduce the depletion of CAR-T cells and increase their persistence. CAR-T cells lacking the NR4A1, NR4A2 and NR4A3 transcription factors have downregulated expression of PD-1 and TIM3 and stronger antitumor effects (56). CAR-T cells overexpressing c-Jun have increased secretion of IL-2 and IFN-γ an increased the proportion of memory T cells, and prolonged the survival of tumor-bearing mice (57).

In the report, the persistence of CAR-T cells was positively correlated with the number of memory T cells. Researchers have found that weak TCR signaling favors memory T cell differentiation, while strong TCR signaling promotes differentiation into effector T cell subsets. The amount and type of immunoreceptor tyrosine-based activation motif (ITAM) domains in CD3 and the TCR complex are related to TCR signal intensity (58). Feucht et al. found that knockout of CD3 ITAMs could induce a memory T-cell phenotype, which demonstrating better antitumor ability. The study demonstrated that the CAR-T cell therapy achieved a lasting and complete tumor response (59–61).

In addition, the persistence of CAR-T cells can be improved by changing the length and composition of extracellular spacers. Modifying long IgG4/IgG2-derived spacers in CAR constructs reduced antigen-independent tetanic signaling in anti-GPRC5D CAR-T cell models, thereby increasing specific antigen-dependent CAR activation (62).

In addition, researchers have designed CD22 CAR and CD133 CAR with either a short or long scFv linker, and found that the short scFv CAR (CART22-short or CD133 CAR-short) had excellent cytotoxicity, secreted more IFN-γ, IL2 and TNF-α, resulted in lower expression of exhaustion-associated surface proteins, exhibited significant anti-leukemia activity and improved animal survival. Thus CD22 CAR and CD133 CAR improved the persistence of CAR-T cells *in vivo* (63).

In addition, the persistence of CAR-T cells can be improved by using humanized CAR-T cells instead of mouse CAR-T cells due to poor immune responses related to the antigen binding domain.

These studies suggest that T cell persistence can also be increased *in vivo* by adding domains that regulate transcription factors to the CARs structure or by attenuating TCR signaling in the CARs structure.

In conclusion, first-generation to the fourth-generation CAR structures have mainly been developed with costimulatory signaling molecules and cytokine secretion in mind, while there have been few studies of the signal strength of the TCR itself. In the future, the relationship between TCR signal strength and persistence of CAR-T cells *in vivo* should be further explored.

## 3 Increasing the proportion of memory T cells in CAR-T cells to improve CAR-T cell persistence

There are many subsets of T cells, each with different proliferative potential. Naive T (TN, CD45RA^+^CCR7^+^CD62L^+^) cells, stem memory T (TSCM, CD45RA^+^CCR7^-^CD62L^+^) cells and central memory T (TCM, CD45RA^-^CCR7^+^CD62L^+^) cells have a higher proliferation potential than effector T cells, and higher proportions of these cells increase the persistence of CAR-T cells *in vivo* (12). Therefore, to increase the persistence of CAR-T cells, it is necessary to increase the proportion of memory T cells in CAR-T cells, which can be achieved by the following four methods: (1) preventing T cell differentiation, (2) reprogramming T cells into terminally differentiated cells, (3) shortening the culture time of CAR-T cells, (4) delaying CAR-T cell senescence.

### 3.1 Preventing T cell differentiation

#### 3.1.1 Cytokines

In a study of CD19 CAR-T cell therapy for chronic lymphocytic leukemia (CLL), Fraietta.et. al. found that CAR-T cells in CLL patients with complete remission were rich in the markers IL-6/STAT3, which increased the proportion of memory T cells(CD27^+^CD45RO^-^ CD8^+^) (13).

It is well known that T cells can differentiate into effector T cells under the action of self-secreted IL-2 (64), but IL-2 secretion is not good for the persistence of T cells *in vivo*. Therefore, Fei Mo et al. designed a IL-2 partial agonist (H9T) that can promote the stemness of CD8+ T cells through the STAT5 signaling pathway (65).

IL-7 promotes the expansion of primitive T cells and maintains the TCM T cell pool by increasing expression of the anti-apoptotic molecules Bcl-2. Studies have shown that CD19 CAR-T cells expressing IL-7R have a better ability to expand *in vivo* and resist apoptosis, thus improving the persistence of CAR-T cells *in vivo* (65).

IL-15 can regulate the homeostatic proliferation of memory CD8+ T cells, and can improve the lytic ability of memory CD8 + T cells by increasing the expression of perforin, granzyme B and IFN-γ. The survival of memory CD8 + T cells can also be promoted by increasing the expression of the anti-apoptotic molecules Bcl-2 and Bcl-X L as well as that of the costimulatory molecule 4-1BB (17). The addition of a IL-15 domain into the CAR improved the survival rate of GD2 CAR-T cells and prolong antitumor activity (66).

Recently, it was found that culturing naive CD19 CAR-T cells with IL-7 and IL-15 promoted their differentiation into CAR-TSCM cells and showed antitumor activity *in vitro* and in mouse models (67).

IL-21 can not only direct CD8+ T cells to express L-selectin during secondary stimulation to promote the secondary proliferation of CAR-T cells (68), but also inhibit Treg cells (14).

The combined expression of IL-15 and IL-21 maintained the expression of T cytokine 1 (TCF-1), a transcription factor essential for T cell development and survival. Research shows that Gpc3 CAR-T cells coexpressing IL-15 and IL-21 enriched poorly differentiated T cells pools, exhibited the strongest peak amplification and persistence *in vivo*, and mediated increased tumor control and survival in hepatocellular carcinoma (HCC) tumor-bearing mice compared to cytokines alone or controls (69).The above studies show that the expression of IL-7, IL-15 and IL-21 can promote the expansion of memory CD8+ cells and improve their antitumor activity.

#### 3.1.2 Medicine

Many small molecule drugs can enhance the effect of T cell therapy by regulating TCR, cytokines, costimulatory ions and growth factor receptors to alter T cell differentiation. For example, the mTOR inhibitor rapamycin can promote the formation of memory CD8+ T cells by regulating the expression of the transcription factors T-bet and eomesodermin, thereby enhancing antitumor function (70). In addition, metformin, a drug forn anti- type 2 diabetes drug, can restore CD8+ memory cells and enhance survival in TRAF6-deficient mice by regulating AMPK-activation and mitochondrial fatty acid oxidation (FAO) (71). Besides, immunomodulatory drugs (IMiDs; lenalidomide and pomalieradomide), which bind to the bispecific T-cell engager molecule AMG 701, can not only induce T-cell-dependent cytotoxicity (TDCC) in multiple myeloma (MM) cells, but also improve the proportion of TSCM cells *in vitro* and have long-lasting antitumor effects in MM mice (72). Furthermore, GSK3β inhibitors can promote the self-renewal of memory T cells through upregulation of the Wnt/β-catenin pathway (60). A small molecule inhibitor of lactate dehydrogenase (LDH) can inhibit aerobic glycolysis and maintain a metabolically quiescent state, and can inhibit CD8+ T cell depletion by regulating the mRNA expression of members of the NR4A family of nuclear receptors, as well as Prdm1 and Xbp1, and can promote TSCM cell production and produce powerful antitumor effects in collaboration with IL-21 (73).

These studies suggested that small-molecule drugs can regulate the proliferation and differentiation of memory T cells through different mechanisms. Therefore, these small-molecule drugs can be used in combination with CAR-T cell therapy to prolong the persistence of CAR-T cells *in vivo*.

#### 3.1.3 Proportion of CD4+ T cells and CD8+ T cells in injected CAR-T cells

The ratio of CD4+ and CD8+ T cells is highly variable in patients with B-ALL and NHL, and studies have shown that the injection a 1:1 ratio of CD4 to CD8 CAR-T cells can ameliorate this phenomenon, resulting in predictable polyclonal proliferation of CD4 + and CD8 + T cells in patients, thereby increasing the persistence of CAR-T cells (15, 16).

### 3.2 Reprogramming terminally differentiated T cells

In response to the constant stimulation of tumor cells, CAR-T cells injected *in vivo* continued to differentiate into tumor-infiltrating lymphocytes (TILs), going down the terminal differentiation a path of terminal differentiation and no longer providing sustained and effective antitumor effects. Therefore, the persistence of CAR-T cells could be improved by modifying the terminal differentiation status of CAR-T cells using gene editing. Studies have shown that TILs can be forced into induced pluripotent stem (IPS) cells capable of maintaining the variables (V), diversity (D), and junction (J) rearrangement region of the TCR chain *via* expression of the SOX2, OCT4, MYC and KLF4 transcription factors. However, this approach is inefficient, and thus a method to reverse terminal differentiation by forcing late differentiated terminal effector T (TEF) cells to express TN and TSCM cell related transcription factors through direct reprogramming has also been developed (18, 19). Studies have used MEK1/2 inhibition (MEKi) to reprogram CD8+ T cells into TSCM cells, so that they can self-renew, longer existence *in vivo*, and stronger antitumor effect (74). It has also been reported that the NOTCH-FOXM1 axis can be reprogrammed by mitochondrial metabolism to transform traditional human CAR-T cells into TSCM-like CAR-T cells, thus increasing longevity and achieving greater antitumor potential *in vivo* (75).

### 3.3 Shortening the culture time of CAR-T cells

Currently, most T cell engineering regimens typically amplify T cells *in vitro* for 9 to 14 days. CD19 CAR-T cells show less differentiation and improved effector function when harvested from cultures at earlier time points (day 3 or day 5) than at later time points (day 9) *in vitro*. The results of studies in mouse models have also indicated that CAR-T cells that are cultured for a shorter time have better therapeutic potential (20).

### 3.4 Delaying CAR-T cell senescence by preventing telomere loss

Transient addition of a modified mRNA encoding telomerase reverse transcriptase to the CAR structure of CD19 CAR-T cells increases telomerase activity in these cells. Compared to conventional CD19 CAR-T cells, these cells have increased proliferation, enhanced persistence and improved antitumor ability (21).

Therefore, there are many ways to promote the maintenance of CAR-T cells: adding specific cytokines such as IL-5, IL-7, and IL-21 to CAR-T cell therapy or adding small-molecule drugs; reprogramming terminally differentiated CAR-T cells; shortening the culture time of CAR-T cells *in vitro*; and preventing the loss of telomerase in CAR-T cells to slow aging. In the future, we can continue to explore ways to help CAR-T cells differentiate into TSCM cells with these four strategies.

## 4 Improving the TME to increase CAR-T cell persistence

CAR-T cell therapy has shown remarkable therapeutic efficacy against leukemia and lymphoma. However, over time, T cell exhaustion appears in the TME. This is related to the overexpression of inhibitory molecules such as PD-1, TIM-3, CTLA-4 and LAG-3 by dysfunctional T cells, as well as cytokines in the TME.

### 4.1 Overcoming checkpoint inhibition to increase CAR-T cell persistence

PD-1, LAG3 and CTLA-4 are common immune checkpoint molecules in the TME. There are some common ways to inhibit these molecules. The first way is to use checkpoint inhibitors (nivolumab, atezolizumab and pembrolizumab) combined with CAR-T cells (23, 76, 77). The second way is to construct CAR-T cells that can directly express immune checkpoint inhibitors (PD-1/CD28 CD19 CAR-T cells) (78–80). The third way is to utilize gene editing technology (CRISPR-Cas9, TALEN) to directly knock out immune checkpoint related genes (76, 81, 82). All of these approaches have demonstrated better T cell proliferation, cytokine production, killing capabilities and persistence *in vitro* than CAR-T cells alone.

In addition to the use of immune checkpoint inhibitors and the construction of novel CAR-T cells by knockout of immune checkpoint genes using gene editing methods, the expression of immune checkpoint molecules can also be regulated by transcription factors (BATF, NFAT, AP-1, and downstream molecules).

In the TME, CAR-T cells overexpressing BATF have increased expansion capacity and cytotoxicity, produce more cytokines and granzymes, prevent T cell depletion and reduce the secretion of the depletion-related transcription factor thymocyte selection-associated HMG BOX (TOX) (83). The high-mobility group (HMG)-box transcription factors (TOX and TOX2) and the NR4A family of orphan nuclear receptors are downstream targets of the transcription factor NFAT. Inhibiting the expression of these cytokines increases the expression of cytokines in CAR-TILs while decreasing the expression of inhibitory receptors. It also has the advantages of inhibiting tumor growth and prolonging the survival of tumor-bearing mice (66, 84). Deficiency of c-Jun transcription factors of the AP-1 family is associated with T cell depletion. Therefore, CAR-T cells overexpressing c-Jun show enhanced expansion potential, reduced terminal differentiation, and improved antitumor efficacy (57).

### 4.2 Utilizing cytokine signaling to increase CAR-T cell persistence

IL-1 is a major proinflammatory cytokine in the TME and is involved in the development and invasion of several tumors (102). In chronic myeloid leukemia (CML), recombinant antibodies targeting IL-1 receptor antagonist (IL-1RA) and IL-1 receptor accessory protein (IL1RAP) can block IL-1 signaling in CML leukemia stem cells (LSCs) and inhibit their growth (85, 86). The combination of IL-1 signaling blockade with a tyrosine kinase inhibitor (TKI) significantly inhibited LSCs growth compared to the TKI alone. In acute myeloid leukemia (AML) and CML, blocking IL-1 signaling (for example, with anti-IL-1RA or anti-IL1RAP antibodies, IRAK1/4 inhibitors, IL-11/4 inhibitors, AP antibodies) eliminates LSCs in the TME, thereby preventing recurrence in patients (85–88).

IL-4 can induce apoptosis of AML cells *in vitro* and *in vivo* through Caspase-3 activation and STAT6 phosphorylation, and can also mediate P53-dependent apoptosis *via* IL-4 on LICs through the endogenous CyPG-PPARγ axis, which is a negative regulator of normal AML cells (89–91).

IL-6 family cytokines are defined as those that use the common signaling receptor subunit glycoprotein 130 kDa (gp130), which is associated BCR/ABL activity in the TME (92). Disrupting IL-6 paracrine signaling of can inhibit the activity of BCR/ABL, and IL-6 inhibitors combined with PD-1 inhibitors can have an antitumor effect, thus contributing to the clearance of cancer cells in the CML TME (93, 94). In CAR-T therapy, IL-6/STAT3 blocked reduced CAR-T cell proliferation (55).

IL-10 is a V-shaped homodimer that can be generated by multiple cell types, and IL-10 has important effects on the TME (103). In the CLL TME, IL-10 is highly expressed, which disrupts the synergy between NFAT and AP-1 through IL-10R-STAT3 signaling, thereby increasing the expression of PD-1 on CD8+ T cells and preventing T cells from playing an antitumor role (95, 96). CAR-T cells targeting the interleukin-10 receptor (IL-10R) also have strong tumor cytotoxicity in AML treatment (97).

IL-12 is a proinflammatory cytokine with strong tumor inhibitory activity that not only stimulates T cells to secrete IFN-γ to enhance the cytotoxicity of CAR-T cells, but also generates resistance to Treg cells effects, reshaping the TME in an IFN-γ-dependent manner (48). Autocrine IL-12 with CAR-T cells in the TME significantly improved the killing effect of CAR-T cells and achieved long-lasting antitumor effects (49–51).

In the TME, studies have shown that the secretion of IL-21 can increase memory and affect CD8+ T cells, inhibit Treg cells, and can cooperate with immune checkpoint inhibitors to improve the depletion of T cells (14, 24, 69, 98).

TGF-β is a common cytokine in the TME and is secreted by malignant cells and their stroma. It promotes tumor growth and metastasis, and potently inhibits APCs and the growth and effector function of T cells and NK cells. TGF-β interacts with TGF-β receptor I (TGF-βRI) or TGF-βII to form type I and type II receptor dimers, resulting in phosphorylation of TGF-β RI and endowing it with the ability to phosphorylate Smad2 and Smad3, which then enter the nucleus to interact with transcription factors. On the one hand, TGF-β can regulate cell growth and inhibit tumor-specific cellular immunity, on the other hand, TGF-βcan inhibit the effects of perforin, granzyme A, granzyme B, Fas ligand and IFN-γ, and inhibit the cytotoxicity of CTLs (104). In Hodgkin’s lymphoma, depletion of endogenous TGF-negative EBV-positive Hodgkin’s CAR-T cells using the CRISPR/Cas9 technique reduced induced Treg transformation and prevented CAR-T cell depletion (99). The specific kinase inhibitor SD-208 can also be administered to block TGF-β-receptor signaling, synergizing the antitumor effects in the TME with CAR-T cells in the TME (100) (**Table 1**).

**Table 1:** The role of cytokines in tumor microenvironment.

Cytokines	Application in tumor microenvironment
IL-1	Blocking IL-1 signaling in CML and AML can inhibit tumor cell proliferation through ADCC-mediated kill sensitivity
IL-4	Inducing apoptosis of AML cells in vitro and in vivo through caspase-3 activation, STAT6 phosphorylation and endogenous CyPGs-PPARγ axis
IL-6	Playing an antitumor role by destroying the paracrine signaling and combining with PD-1 inhibitors
IL-10	Blocking IL-10R-STAT3 signaling can regulate the synergy between NFAT and AP-1
IL-12	Secreting IFN-γ to enhance the cytotoxicity of CAR-T cells, but also generates resistance to Treg cells
IL-21	Promoting the production of memory and effector CD8 ^+^ T cells, inhibiting the growth of Treg cells, and cooperating with immune checkpoint inhibitors to improve the depletion phenomenon in tumor microenvironment
TGF-ß	Blocking TGF-β-receptor II signaling can induced Treg transformation and preventing CAR-T cell depletion

In summary, there are many factors in the TME that inhibit the function of CAR-T cells, such as immunosuppressive molecules, inhibitory cytokines, and Treg cells. Ways to optimize the TME for *via* these factors and improve the persistence of CAR-T cells *in vivo*.

## 5 Concluding remarks

This review focuses on ways to increase the persistence of CAR-T cells *in vivo* by modifying the structure of CAR-T cells, increasing the proportion of memory CAR-T cells, and improving the TME.

Although memory T cells are required in CAR-T cell therapy to increase their persistence *in vivo*, terminally differentiated effector T cells are still required to kill tumor cells. Therefore, how to increase the proportion of stem cells while ensuring the proportion of effector T cells needed to kill tumor is a problem worth thinking about.

In the TME, the expression of inhibitory immune checkpoint proteins on the surface of CAR-T cells was increased, thereby affecting the function of CAR-T cells. The current research focuses on the suppression of immune checkpoint proteins, including the use of immune checkpoint inhibitors, the construction of suppressant-producing CAR-T cells and the construction of CAR-T cells that knock out the genes related to the immune checkpoint proteins. The first two methods are to directly inhibit immune checkpoint proteins, while the latter method is to directly block the expression of immune checkpoint molecules at the gene level. The knockout of related genes using gene editing technology (CRISPR-Cas9, TALEN) can fundamentally reduce the expression of immunosuppressive molecules, which will be a popular method in the future immunotherapy.

Gene editing technology can not only knock out the genes associated with immune checkpoints, but also the genes associated with inhibitory cytokine secretion. It can also insert genes regulated to memory T cells. Therefore, the structure of CARs can be modified by this technology in many aspects to enhance the persistence of CAR-T structure. However, the stability and security of these domains should also be considered.

In addition, the persistence of CAR-T cells *in vivo* is not always better. For example, CD19 CAR-T cells can target both malignant cells and non-normal B cells. If the persistence of CAR-T cells is increased, it will have adverse effects on normal B cells in the patient. While attention should be paid to the persistence of CAR-T cells *in vivo*, treatment side effects (such as CRS and neurotoxicity) should also be paid. In the future, there is an urgent need to observe the side effects and safety of novel CAR-T cell therapies in clinical trials to determine whether they can be safely applied to clinical treatment.

## Author contributions

Conceptualization, YK, LT, YY, and XZ; writing—original draft preparation, YK and LT; writing—review and editing, YK, LT, YY, QL, and XZ; supervision, YK, LT, YY, QL, and XZ. All authors contributed to the article and approved the submitted version.
